# Exploratory Analysis of MicroRNA Alterations in a Neurodevelopmental Mouse Model for Autism Spectrum Disorder and Schizophrenia

**DOI:** 10.3390/ijms25052786

**Published:** 2024-02-28

**Authors:** Susana García-Cerro, Ana Gómez-Garrido, Gonçalo Garcia, Benedicto Crespo-Facorro, Dora Brites

**Affiliations:** 1 Translational Psychiatry Group, Ibis-Biomedicine Institute of Sevilla-CSIC, Manuel Siurot AV, 41013 Seville, Spain; agarrido-ibis@us.es; 2 Spanish Network for Research in Mental Health (CIBERSAM), Monforte de Lemos AV, 3-5, 28029 Madrid, Spain; 3 Neuroinflammation, Signaling and Neuroregeneration Lab, Research Institute for Medicines (iMed.ULisboa), Faculty of Pharmacy, Universidade de Lisboa, 1649-003 Lisbon, Portugal; ggarcia@campus.ul.pt (G.G.); dbrites@ff.ulisboa.pt (D.B.); 4 Department of Pharmaceutical Sciences and Medicines, Faculty of Pharmacy, Universidade de Lisboa, 1649-003 Lisbon, Portugal; 5 Mental Health Unit, Virgen del Rocio University Hospital, Manuel Siurot AV, 41013 Seville, Spain; 6 Department of Psychiatry, Faculty of Medicine, University of Seville, Sánchez Pizjuán AV, 41013 Seville, Spain

**Keywords:** ketamine-treated mouse pups, autism spectrum disorder, schizophrenia, prefrontal cortex, miRNA, sexual dimorphism

## Abstract

MicroRNAs (miRNAs) play a crucial role in the regulation of gene expression levels and have been implicated in the pathogenesis of autism spectrum disorder (ASD) and schizophrenia (SCZ). In this study, we examined the adult expression profiles of specific miRNAs in the prefrontal cortex (PFC) of a neurodevelopmental mouse model for ASD and SCZ that mimics perinatal pathology, such as NMDA receptor hypofunction, and exhibits behavioral and neurophysiological phenotypes related to these disorders during adulthood. To model the early neuropathogenesis of the disorders, mouse pups were administered subcutaneously with ketamine (30 mg/Kg) at postnatal days 7, 9, and 11. We focused on a set of miRNAs most frequently altered in ASD (miR-451a and miR-486-3p) and in SCZ (miR-132-3p and miR-137-3p) according to human studies. Additionally, we explored miRNAs whose alterations have been identified in both disorders (miR-21-5p, miR-92a-2-5p, miR-144-3p, and miR-146a-5p). We placed particular emphasis on studying the sexual dimorphism in the dynamics of these miRNAs. Our findings revealed significant alterations in the PFC of this ASD- and SCZ-like mouse model. Specifically, we observed upregulated miR-451a and downregulated miR-137-3p. Furthermore, we identified sexual dimorphism in the expression of miR-132-3p, miR-137-3p, and miR-92a-2-5p. From a translational perspective, our results emphasize the potential involvement of miR-92a-2-5p, miR-132-3p, miR-137-3p, and miR-451a in the pathophysiology of ASD and SCZ and strengthen their potential as biomarkers and therapeutic targets of such disorders.

## 1. Introduction

Participation of miRNAs in essential neurodevelopmental processes, brain plasticity pathways, learning and memory functions, and various other crucial brain mechanisms is well documented [1,2].

miRNAs, composed of 20–24 nucleotides, constitute a major class of small non-coding RNAs and play a crucial role as post-transcriptional regulators, influencing gene expression by downregulating or upregulating protein synthesis or promoting mRNA degradation [3,4,5]. The complexity of miRNA-mediated gene regulation is highlighted by a single miRNA’s ability to regulate hundreds of proteins, and multiple miRNAs dynamically regulating many mRNAs [6]. Consequently, dysregulation of miRNAs is a common feature in neurodevelopmental, neurodegenerative, and psychiatric disorders [7,8,9,10,11], making them potential biomarkers for diagnosis and prognosis in conditions like autism spectrum disorder (ASD) or schizophrenia (SCZ) [12,13].

ASD and SCZ, chronic and debilitating disorders with profound effects on individuals and families, exhibit distinct clinical profiles [14,15]. ASD, a neurodevelopmental condition, typically emerges before age three, affecting 1 to 2% of children, and is characterized by challenges in social interaction, communication, and repetitive behaviors [16,17]. In contrast, SCZ, an adult-onset psychiatric disorder diagnosed typically between ages 16 and 30, has a lifetime prevalence of approximately 1%, and is marked by symptoms like hallucinations, delusions, disorganized speech or behavior, apathy, and emotional unresponsiveness [18,19]. Despite their clinical differences, both disorders share a neurodevelopmental origin, with research indicating overlapping relationships and commonalities in underlying biological mechanisms [20,21,22].

Studies suggest that the neurodevelopmental origins of ASD and SCZ disorders may involve the disruption of inhibitory circuits, leading to an imbalance in excitatory and inhibitory (E/I) brain activity [20,23]. Parvalbumin interneurons (PVs) play a key role in modulating the activity of excitatory pyramidal neurons, contributing to dynamic E/I balance maintenance and cortical synchronization [24,25,26]. Genetically determined early in brain development, the disruption of GABAergic interneurons, including PVs, during development is hypothesized to contribute to the complex scenario observed in individuals with ASD or SCZ [27,28]. Indeed, it is established that PVs are primarily impacted in both disorders [29,30,31].

To model this neurodevelopmental pathogenesis in mice, the non-competitive NMDA receptor antagonist ketamine (30 mg/kg) was administered to mouse pups during postnatal days (PNDs) 7, 9, and 11, a critical postnatal period for PVs’ development [32,33,34]. This results in PV and GABAergic circuitry disruption and E/I disbalance in adulthood [35,36,37], and mirroring in mice behavioral phenotypes observed in ASD and SCZ, such as repetitive behavior, social deficits, reduced prepulse inhibition (PPI) of the startle response, and impaired cognitive flexibility [38,39,40,41].

Given the limited understanding of miRNA roles in ASD and SCZ disorders, and their potential as reliable biomarkers, as well as the scarcity of murine models replicating relevant phenotypes, our study explores persistent miRNA dysregulation in the PFC of a neurodevelopmental mouse model. We comprehensively assessed a set of promising miRNAs, which included some of the most frequently dysregulated miRNAs in both ASD and SCZ individuals. Specifically, we examined miR-451a and miR-486-3p that are most frequently altered in individuals with ASD [42], and miR-132-3p and miR-137-3p, known to be commonly altered in individuals with SCZ [12,43]. Additionally, we explored miR-21-5p, miR-92a-2-5p, miR-144-3p, and miR-146a-5p, all of which exhibit dysregulation in both ASD and SCZ individuals [12,42,44,45,46,47]. Furthermore, we placed a particular emphasis on examining these variations within the context of sexual dimorphism.

## 2. Results

### 2.1. ASD-Linked miR-451a and miR-486-3p Dynamics in the Early Ketamine-Treated Neurodevelopmental Model

Studies of miRNA expression profiles in individuals with ASD highlighted miR-451a and miR-486-3p among the most frequently altered miRNAs [42]. Particularly, miR-451a was shown to be consistently increased in the postmortem brain tissue from ASD patients, as well as in lymphoblastoid cell lines, serum, or saliva [48,49,50,51,52]. In what concerns miR-486-3p, it was only found to be downregulated in serum and blood samples from individuals with ASD [53,54,55]. In this context, we examined the expression of miR-451a and miR-486-3p in the PFC of the early ketamine-treated mice.

**Figure 1:**
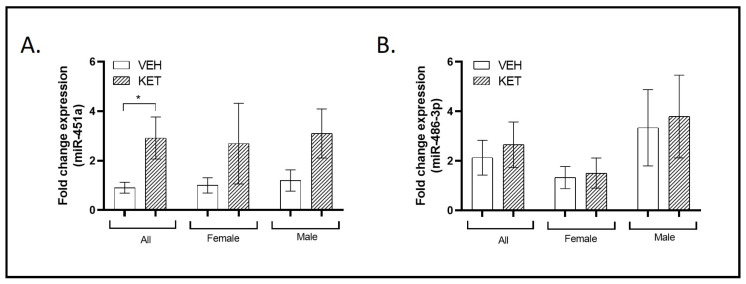
Expression profiles of miRNAs typically associated with ASD in the PFC of the neurodevelopmental murine model. RT-qPCR analysis was conducted to determine miR-451a and miR-486-3p expression levels. Sexual dimorphism was also investigated, and data stratified by gender are shown. (**A**) A significant overexpression of miR-451a was observed in the pharmacological mouse model when compared to the vehicle-treated group (n = 19). (**B**) Ketamine treatment did not affect the miR-486-3p expression levels (n = 20). (**A**,**B**) No significant differences were observed between the sexes (females: miR-451a, n = 9; miR-486-3p, n = 11; males: miR-451a: n = 10; miR-486-3p: n = 9). Results in (**A**,**B**) are mean ± SEM and are expressed as the fold change. Statistical significance was determined using the Student’s *t*-test. * *p* < 0.05, ketamine- vs. vehicle-treated experimental group. Abbreviations: ASD, autism spectrum disorder; PFC, prefrontal cortex; NMDA, N-methyl-D-aspartate; miRNA(miR), microRNA; RT-qPCR, real-time quantitative polymerase chain reaction.

Interestingly, a significant overexpression of miR-451a was observed when compared to the vehicle-treated group (*p* = 0.048; Figure 1A). However, as shown in Figure 1A, no significant differences were found by sex, though the initial tendency towards overexpression was maintained.

In the case of miR-486-3p, early NMDA receptor antagonism had no effect on its expression levels in the PFC when we analyzed the total animals per group or explored possible sexual dimorphism (Figure 1B).

### 2.2. SCZ-Linked miR-132-3p and miR-137-3p Dynamics in the Early Ketamine-Treated Neurodevelopmental Model

Among the miRNAs most characteristically altered in individuals with SCZ, miR-132-3p and miR-137-3p stand out. Increased or decreased levels of miR-132-3p transcript levels have been observed not only in postmortem PFC tissue [43,56] but also in the plasma of individuals with SCZ [2]. On the other hand, dysregulated miR-137-3p transcript levels in a variety of SCZ tissues and single nucleotide polymorphisms (SNPs) have also been consistently associated with an increased risk of SCZ [12]. As this miRNA is one of the most strongly associated SCZ risk loci identified to date, it is also one of the most extensively characterized at the biological and functional levels [57]. Therefore, we evaluated the dynamics of these miRNAs in the early ketamine-treated mice.

**Figure 2:**
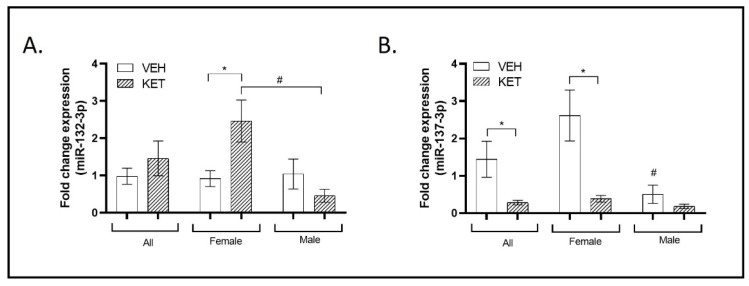
Expression profiles of miRNAs typically associated with SCZ in the PFC of the neurodevelopmental murine model. RT-qPCR was conducted to determine miR-132-3p and miR-137-3p expression levels. Sexual dimorphism was also investigated, and data stratified by gender are shown. (**A**) The expression levels of miR-132-3p in the perinatal ketamine-treated animals did not significantly differ from those treated with the vehicle when considering all animals (n = 18). However, when stratified by sex, a significant sexual dimorphism was observed. Specifically, female animals treated with ketamine exhibited a significant overexpression of miR-132-3p transcript (n = 10). Additionally, a significant opposite trend in the expression levels of miR-132-3p was observed between the male and female ketamine groups (n = 9). (**B**) The expression levels of miR-137-3p were significantly lower in the ketamine-induced murine model. This trend was observed exclusively within the female experimental groups (n = 17). Additionally, significant sexual dimorphism was identified when comparing miR-137-3p expression levels between female and male vehicle-treated animals (n = 9). Results in (**A**,**B**) are mean ± SEM and are expressed as the fold change. Statistical significance was determined using the Student’s *t*-test. * *p* < 0.05, ketamine- vs. vehicle-treated experimental group; # *p* < 0.05, female vs. male experimental groups. Abbreviations: SCZ, schizophrenia; PFC, prefrontal cortex; NMDA, N-methyl-D-aspartate; miRNA(miR), microRNA; RT-qPCR, real-time quantitative polymerase chain reaction.

Taking a general perspective, the expression levels of miR-132-3p in the perinatal ketamine-treated animals did not significantly differ from those treated with the vehicle (Figure 2A). Nevertheless, upon analyzing the data while considering possible variations linked to the sex of the animals, a notable sexual dimorphism became evident. The levels of miR-132-3p varied between the experimental groups when considering only female animals. In this specific case, females treated with ketamine exhibited an overexpression of miR-132-3p transcript compared to vehicle-treated mice (*p* = 0.026; Figure 2A). Conversely, no significant differences were observed between male groups. Notably, when comparing both female and male mice treated with ketamine, higher and lower levels of miR-132-3p, respectively, were revealed (*p* = 0.014; Figure 2A).

Regarding miR-137-3p, its expression levels were markedly decreased in the PFC of the ketamine-treated mice compared to vehicle-treated animals (*p* = 0.043; Figure 2B). This same trend was observed when analyzing only the female experimental groups. Female ketamine-treated animals exhibited a suggestive downregulation of miR-137-3p small RNA compared to females treated with the vehicle (*p* = 0.018; Figure 2B). However, when comparing only the male groups of mice, the levels of miR-137-3p expression in ketamine-treated mice did not significantly differ from those in vehicle-treated animals. Furthermore, sexual dimorphism associated with miR-137-3p was found when comparing its expression levels in the PFC of vehicle-treated animals, with significantly higher levels observed in the female group (*p* = 0.015; Figure 2B).

### 2.3. ASD and SCZ-Linked miRNAs’ Dynamics in the Early Ketamine-Treated Neurodevelopmental Model

As mentioned earlier, ASD and SCZ share some clinical similarities, which are hypothesized to be linked to the same biological basis, particularly in terms of E/I imbalance [58] and PV dysfunction [23,27,59]. Building on this concept, we investigated the expression levels of miR-21-5p, miR-92a-2-5p, miR-144-3p, and miR-146a-5p in the PFC of the early ketamine-treated mice.

**Figure 3:**
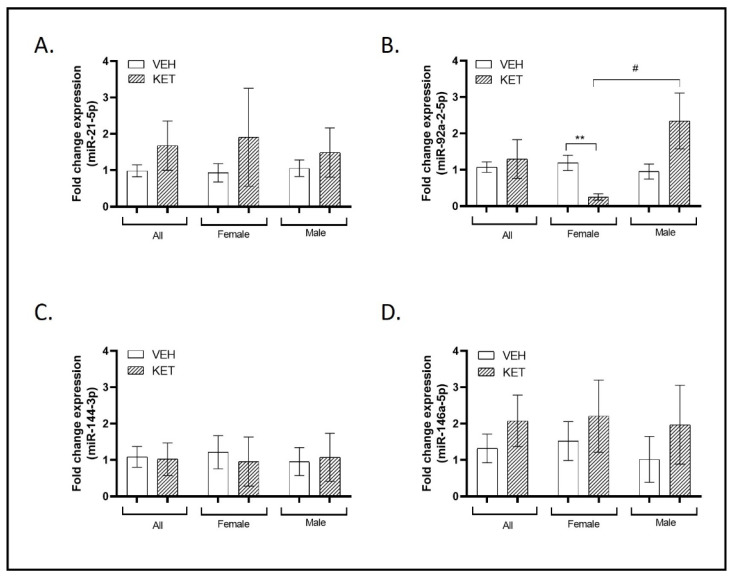
Expression profiles of miRNAs associated with both ASD and SCZ in the PFC of the neurodevelopmental murine model. RT-qPCR was conducted to determine miR-21-5p, miR-92a-2-5p, miR-144-3p, and miR-146a-5p expression levels. Sexual dimorphism was also investigated, and data stratified by gender are shown. (**A**) A trend to present enhanced miR-21-5p expression was found in mice subjected to ketamine early treatment, although this effect did not reach statistical significance (n = 20). (**B**) The expression of miR-92a-2-5p remained unaltered with early ketamine treatment in mice compared to those treated with the vehicle when considering all animals (n = 19). However, when stratified by sex, significant sexual dimorphism was observed. Specifically, female ketamine-treated animals exhibited a significant reduction in the expression of this miRNA in comparison to female vehicle-treated animals (n = 9). Significant sexual dimorphism was also identified when comparing miR-92a-2-5p expression levels between female and male ketamine-treated animals (n = 10). (**C**) Ketamine treatment did not affect the miR-144-3p expression levels, and no significant differences were observed between the sexes (n = 21). (**D**) A non-significant trend toward increased miR-146a-5p expression was observed in the ketamine-treated group when considering all animals (n = 21) and when stratified by gender (females: n = 11; males: n = 10). Results in (**A**–**D**) are mean ± SEM and are expressed as the fold change. Statistical significance was determined using the Student’s *t*-test. ** *p* < 0.01, ketamine- vs. vehicle-treated experimental group; # *p* < 0.05, female vs. male experimental groups. Abbreviations: SCZ, schizophrenia; ASD, autism spectrum disorder; PFC, prefrontal cortex; NMDA, N-methyl-D-aspartate.

Decreased levels of miR-21-5p have been documented in peripheral blood mononuclear cells (PBMCs) from SCZ patients [44,60,61] and in the PFC tissue of individuals with ASD [50]. When we analyzed the levels of this miRNA in the PFC of the different experimental groups of animals, a tendency to present enhanced miR-21-5p expression was found in mice subjected to ketamine early treatment, although this effect did not reach statistical significance. This non-significant tendency was maintained when male and female groups were studied separately (Figure 3A).

Elevated expression of miR-92a-2-5p has been observed in both ASD and SCZ blood compared to control samples, as well as in ASD lymphoblastoid cell lines [49,52,62,63,64]. The PFC expression of miR-92a-2-5p remained unaltered with early ketamine treatment in mice compared to those treated with the vehicle when data were not stratified (Figure 3B). However, we detected sexual dimorphism in relation to this miRNA when comparing exclusively female animals. Specifically, female ketamine-treated animals exhibited a significant reduction in the expression of this miRNA in comparison to females treated with the vehicle (*p* = 0.007; see Figure 3B). Furthermore, we observed an opposing effect of ketamine administration between individuals of different sexes in the levels of miR-92a-2-5p, as its expression displayed a significant countertrend (*p* = 0.035; Figure 3B).

Altered miR-144-3p expression has been noted in various samples from individuals with ASD, including brain tissue, serum, and peripheral blood [50,65,66], as well as in the peripheral blood of individuals with SCZ [67]. In this case, the treatment did not affect its levels and no remarkable differences were observed between sexes (Figure 3C).

miR-146a-5p concludes our examination of small non-coding RNAs in this section. SNPs in this miRNA have been associated with chronic schizophrenia in patients [68]. Moreover, overexpressed levels have been detected in lymphoblastoid cell lines and saliva of individuals with ASD [50,51,63,69]. When we compared the levels of this transcript between both groups of animals, a non-significant trend toward higher miR-146a-5p expression was observed in the ketamine-treated group. Additionally, miR-146a-5p levels were not influenced by sexual dimorphism (Figure 3D).

### 2.4. Correlation of miRNA Expression and Common Targets within the Investigated Set

**Figure 4:**
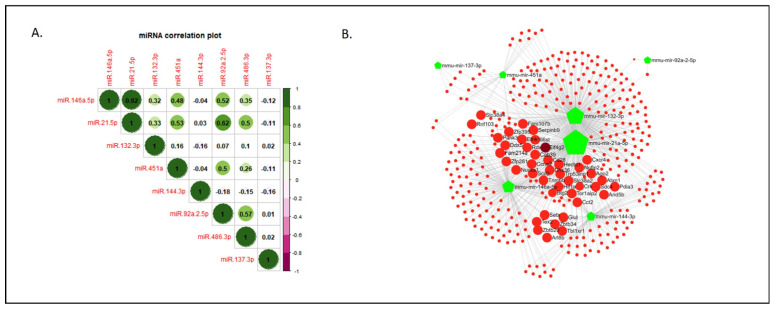
Correlation of miRNA expression within the investigated set of miRNAs in the neurodevelopmental murine model and miRNA-Targeting Network for the investigated set of miRNAs. (**A**) The correlation matrix illustrates the pairwise relationships between the expression levels of the eight investigated miRNAs using Pearson correlation (r). Positive and negative values of the obtained r are depicted. (**B**) Mouse (*Mus musculus*) miRNA identifiers (IDs), showed as green pentagons, were interrogated in the open-source platform miRNet, as described in Section 4. The analysis revealed a total of 367 nodes (red circles), of which 42 (large red circles) show a degree level from 3 to 5, with a betweenness level of 1. The darker red circle indicates the highest node in the analysis (Eif4G2). Green polygons represent the input miRNAs, with larger sizes indicating more targets. Abbreviations: Eif4G2, Eukaryotic translation initiation factor 4 gamma 2; miRNA(miR), microRNA; NMDA, N-methyl-D-aspartate.

To investigate the potential associations between miRNAs sharing similar functions or likely involved in a common metabolic pathway or biological process, we conducted a correlation analysis using the Pearson correlation coefficient. This involved studying both the vehicle- and ketamine-treated groups. We identified a high positive correlation (r = 0.92) between miR-146a-5p and miR-21-5p. Furthermore, miR-146a-5p exhibited positive correlations with four other miRNAs (refer to Figure 4A), including a significant connection with miR-451a (*p* = 0.016) and miR-92a-2-5p (*p* = 0.009). Interestingly, miR-21-5p displayed positive correlations with the same four miRNAs as miR-146a-5p, in addition to the previously mentioned correlation with miR-146a-5p (see Figure 4A,B). Moreover, miR-21-5p showed a significant association with miR-451a (*p* = 0.008) and miR-92a-2-5p (*p* = 0.001), as well as with miR-486-3p (*p* = 0.012). Finally, similar associations of miR-451a and miR-92a-2-5p with other miRNAs were observed, consistent with the positive correlations among them and with miR-146a-5p, miR-21-5p, and miR-486-5p. These correlations also showed significant associations between them (*p* < 0.05; see Table S1), in addition to the previously mentioned significant associations with miR-146a-5p and miR-21-5p.

To further explore our selected miRNAs, we created a protein targeting network for the miRNAs set in mice (*Mus musculus*) (Figure 4B). The miRNA–protein interaction network revealed interesting target associations among seven miRNAs (more specifically 367 shared nodes), excluding miR-486-3p due to its insufficient targeting interactions with others. Specifically, 42 protein targets, including members of the Insulin-like growth factor (Igf) family, Eukaryotic translation initiation factor 4E (eIF4E) family, Serpin family, Matrix metalloproteinases (Mmp) family, Zinc finger proteins (Zfp) family, or Hypoxia inducible factor 1 subunit alpha (Hif1a) protein, were identified as highly co-regulated by miR-146a-5p and miR-21-5p, along with mir-132-3p, mir-451a, mir-137-3p, or mir-92a-2-5p (Datasets S1 and S2).

## 3. Discussion

Psychiatric disorders exhibit a significant genetic component [70,71,72]. However, this genetic landscape becomes even more complex when we consider intricate regulatory mechanisms, such as miRNA-mediated interactions [73]. Consequently, the latest research in this field is shifting towards a more holistic approach, integrating miRNA-based insights with other biological factors to achieve a more comprehensive understanding [74].

In this study, we specifically focused on a set of miRNAs frequently found to be altered in both ASD and SCZ patients, aiming to investigate miRNA dynamics at the preclinical level. To achieve this, we employed a well-established mouse neurodevelopmental model of ASD and SCZ with short-term NMDA receptor hypofunction [35,36,38,39,40,41,75], which is based on the early administration of ketamine (ketamine-treated mouse pups), with the purpose of investigating a specific group of miRNAs. This set encompasses several non-coding RNAs that are commonly dysregulated in conditions like ASD and SCZ, including miR-144-3p, miR-451a, miR-486-3p, miR-132-3p, miR-137-3p, miR-21-5p, miR-146a-5p, and miR-92a-2-5p. It is important to keep in mind that the selection of the current set of miRNAs examined here was based on the results from human studies. Each selected miRNA exhibited alterations, either in downregulation or overexpression levels, in ASD and/or SCZ individuals. However, in certain cases, such as for miR-132-3p or miR-144-3p, there is controversy regarding the direction of the altered expression. Differences in the study design, tissue examined, and methodologies employed may explain the discrepancies in these findings.

In the context of the present study, mouse pups treated with ketamine exhibited PFC overexpression of miR-451a in adult life. This finding is particularly interesting since a recent meta-analysis conducted by our research group determined that miR-451a is one of the most dysregulated (overexpressed) miRNAs in individuals with ASD, with relevance to clinical practice and being strongly associated with impaired social interaction [42]. Overexpression of this miRNA was associated with elevated oxytocin receptor (OXTR) mRNA expression and is linked to impaired social interaction in individuals diagnosed with ASD [50,76]. Furthermore, genetic disruptions in OXTR have been linked to certain subpopulations of individuals with ASD, including those with Asperger’s syndrome [77]. Therefore, the adult dysregulation of miR-451a found in the neurodevelopmental murine model aligns with the social alterations typically observed in this model [35,38,39,40,41,75]. Additionally, miR-451a upregulation has been predicted to be related to the downregulation of *SLC17A7*, which encodes vesicular glutamate transporter 1 (VGLUT1) protein, suggesting a potential interaction with glutamatergic pathways [78].

Remarkably, miR-451a has been associated with other psychiatric disorders such as major depression disorder (MDD), anxiety, or stress [78,79,80,81,82], and has even been studied as a predictor of response to antidepressant treatment [83]. In fact, several authors have addressed the relationship between this miRNA, glutamatergic pathways, cellular oxidative stress markers, and the Macrophage Migration Inhibitory Factor, which has also been related to redox imbalance in MDD [80,81]. This evidence is not surprising, as accumulated data suggest the importance of the glutamate puzzle in MDD and mood disorders, as well as in ASD or SCZ [84].

Now considering the miR-486-3p expression in the PFC, we did not find any significant alteration in the ketamine-treated mice during adult life. This miRNA was demonstrated to be associated with behavioral and cognitive impairments by regulating the expression of *AT-Rich Interaction Domain 1B* (*ARID1B*) gene. As far as we know, it was only described to be altered in ASD at the level of lymphoblastoid cell lines and serum, and not in the brain [53,54,55].

Next, and in addition to the changes observed in miR-451a, the expression levels of miR-137 were found to be significantly lower in the PFC of the ketamine-treated mice when compared to control animals. Notably, genome-wide association studies (GWAs) have identified SNPs in the human *MIR137* gene locus associated with an increased risk of SCZ [57,85,86,87]. This miRNA is believed to play a key role in the neural differentiation and maturation, as well as in controlling synaptic function by regulating processes such as synaptogenesis, synapse maturation, and synaptic transmission [85]. With this background, it is perhaps not surprising that miR-137 has been reported to be genetically associated with psychiatric disorders such as bipolar disorder (BD), MDD, or intellectual disability [88,89,90,91], although with less clarity than in the case of SCZ.

Interestingly, mice hemizygous for neuronal miR-137 exhibit deficits in synaptic plasticity, social behavior, and learning, in addition to displaying repetitive behaviors [92], mirroring the behavioral phenotype of the mice model used in this study [35,36,38,39,40,41,75]. Furthermore, alterations in miR-137 have also been observed in the pharmacological MK-801 mouse model [93]. Remarkably, the same authors demonstrated that miR-137 targets both Grin2A and Grin2B, which encode the subunits NR2B and NR2A of the NMDA receptor [94] that is crucial for PV functionality.

Research suggests that, while not the primary cause of ASD or SCZ, the adverse effects of oxidative stress significantly contribute to the pathophysiology of these disorders [95,96]. Redox dysregulation has been linked to NMDA receptor hypofunction, neuroinflammation, or alterations in mitochondrial bioenergetics, initiating early “vicious cycles” associated with oxidative stress during neurodevelopment. These processes exacerbate each other, leading to persistent disruptions in the maturation and function of PV-GABAergic neuron circuits [97].

Additionally, Khadimallah et al. (2022) [98] reported that dysregulation of miR-137 levels results in oxidative stress-related changes in PVs, leading to the disruption of autophagy markers such as NIX, FUN14 domain containing 1 (Fundc1), and microtubule-associated protein 1A/1B-light chain 3B (LC3B), as well as the accumulation of damaged mitochondria. Interestingly, the inhibition of NMDA receptors during development using ketamine has been shown to induce oxidative stress alterations [75].

Several studies have indicated that miR-137 plays a protective role against oxidative stress and inflammation [99,100]. Therefore, the lower levels of miR-137 observed in this study may suggest an increased susceptibility to these stressors. Consequently, future studies involving the animal model examined here will be essential for elucidating the functional relationship between PVs and miR-137.

For miR-144-3p, which directly regulates the expression of glutamate decarboxylase 1 (GAD1) and the vesicular GABA transporter (VGAT) [12,101] associated with plasticity signaling pathways [46], no modifications were induced by the ketamine treatment. It is important to note that although miR-144-3p has been associated with ASD and SCZ in the literature, this miRNA is prominently associated with MDD [102]. Similar findings were observed for miR-146a-5p, which has been linked to the proper differentiation of neural stem cells during brain development [103]. When upregulated, it has been shown to increase the uptake of extracellular glutamate, potentially disrupting the homeostasis of glutamate at the synapses, and further impairing synaptic transmission [69,104].

Another miRNA altered in both SCZ and ASD patients is miR-21 [44,60,61]. This miRNA has been found to positively correlate with oxytocin mRNA expression in human subjects and negatively with the levels of OXTR, suggesting a possible mechanism related to social interaction functions [50]. Furthermore, miR-21-5p is known to be involved in glutamate toxicity and synaptic dysfunction [105] and has been described as a critical immune regulator in various pathological contexts [106]. However, we were unable to identify any significant alteration in the ketamine-treated mice.

Sex-related differences in biological processes have the potential to enhance our understanding of the modified physiological mechanisms in individuals with ASD or SCZ, ultimately leading to improved reproducibility and experimental efficiency [107]. In this regard, our research revealed sexual dimorphism in the expression of miR-137-3p, but also in that of miR-132-3p and miR-92a-2-5p, in the ketamine-treated mice. Notably, overexpression of 132-3p was proposed to induce the activation of glial cells [108] and has been linked to post-traumatic stress disorder, MDD, as well as SCZ [109]. On the other hand, miR-92a-2-5p plays a critical role in neuronal neurite outgrowth, neuronal differentiation, and GABAergic neuron maturation [44,55,110]. Although this miRNA is altered in both ASD and SCZ patients, the miR-92a family has also been implicated in anxiety and MDD [79]. Additionally, it is important to keep in mind that miR-92a-2-5p is not conserved between humans and mice (Table 1), and the mouse version of the target sequence was used for this miRNA in this study. While ketamine-treated mice displayed higher levels of miR-132-3p in the female animals, they concomitantly showed lower levels of miR-137-3p. Moreover, the miR-92a-2-5 showed opposing trends from females (reduced values) to males (enhanced values). This is in line with the wide scientific literature showing the association between sex differences and miRNA expression in numerous disorders, including cancer, diabetes mellitus type 2, or ischemic stroke [111,112,113]. Furthermore, sex differences in mental disorders or conditions, including SCZ and ASD, are among the most intriguing and stable findings in psychiatry [114]. A better understanding of the mechanisms behind sex-related differences in miRNA expression and their impact on susceptibility, development, and progression of conditions like ASD or SCZ is then imperative.

Evidence suggests that miRNAs often form clusters that are co-expressed and function collaboratively, regulating various aspects of cellular function such as cellular growth, proliferation, differentiation, or development, among others [115]. To explore potential associations among the expression patterns of eight selected miRNAs, we conducted a correlation analysis in both vehicle- and ketamine-treated mice. Notably, both miR-146a-5p and miR-21-5p values showed correlations with at least five miRNAs. We also investigated common targets for this set of miRNAs in mice, revealing strong interactions among seven out of the eight selected miRNAs, collectively regulating hundreds of common targets. Specifically, proteins associated with the Igf, eIF4E, Mmp, and Zfp families—known for their implications in ASD or SCZ—appeared to be highly shared targets of miRNAs altered in this study, such as miR-21a-5p, miR-146a-5p, miR-451a, miR-132-3p, and miR-92a-2-5p. However, the ASD-associated miR-486-3p showed limited interaction within the network, possibly due to independent regulatory mechanisms.

Studying miRNA dynamics at the preclinical level provides valuable insights for identifying potential common biomarkers with translational implications for ASD and SCZ, aiding in patient stratification and the discovery of new therapeutic targets [116]. Notably, preclinical research has showcased the enduring and reversible effects of miRNA-based therapies [117]. For instance, the efficacy of intravenously administered anti-miR-122 treatment for Hepatitis C has been demonstrated in both primates [118] and humans using subcutaneous injection [119] without significant adverse effects. Additionally, significant benefits were observed with the intrathecal administration of a miR-124-depleted secretome in a mouse model of amyotrophic lateral sclerosis (SOD1-G93A), preventing neurodegeneration and glial dysfunction, along with a marked delay in disease progression [120].

Throughout this discussion, it can be observed that while our study focuses on the miRNAs most frequently dysregulated in individuals with ASD and SCZ, many of the miRNAs discussed are also altered in other psychiatric conditions such as MDD, BD, mood disorders, or intellectual disability. This is not completely surprising, given the polygenic and complex nature of these disorders [121]. In the context of humans, it is crucial to consider that a single miRNA can simultaneously regulate the expression of multiple genetic factors. This emphasizes the impact of the genetic landscape on the specific functions of miRNA in conditions such as ASD and SCZ, as well as in other psychiatric disorders. Additionally, the overlap across psychiatric disorders is also reflected in similar clinical features and related traits [122]. In this regard, while ASD and SCZ exhibit distinct clinical characteristics, they share certain phenotypical similarities [123]. This might reflect a common biological basis [21], such as the developmental disruption of inhibitory circuits and PV functionality, as discussed in this work.

So far, diagnosis of ASD and SCZ relies solely on signs and clinical symptoms. The absence of definitive biomarkers leaves clinical psychiatry vulnerable to undesirable fluctuations in diagnostic and therapeutic decisions [72]. Preclinical investigations, as presented here, contribute to a deeper comprehension of the shared biological mechanisms underlying these conditions and potentially facilitate the development of novel therapeutic and diagnostic strategies. Additionally, animal models offer an opportunity to study miRNA expression patterns in specific brain regions and associate them with different behaviors [124]. In the context of the murine model with short-term NMDA receptor hypofunction, we observed alterations in several miRNAs, highlighting their relevance to this specific biological scenario. In most cases, critical miRNAs for brain function are highly conserved between mice and humans [125]. Therefore, murine models serve as a valuable tool for identifying novel miRNAs with potential relevance to physiological phenotypes associated with ASD and SCZ. Our study represents a preclinical exploratory analysis, and further substantial efforts are required to elucidate specific mechanisms of action involving behavioral, neuromorphological, cellular, and molecular alterations related to ASD and SCZ. This includes impairments in PVs, E/I balance, or cognitive and social behavior in the neurodevelopmental ASD- and SCZ-like mice model examined here. Despite these challenges, miRNAs offer a fresh and broad perspective as potential biomarkers for diagnosis and prognosis, presenting an intriguing and potentially novel therapy approach for a wide range of human disorders, including SCZ and ASD. To advance this goal, we have recently initiated the recruitment of a human cohort comprising individuals with ASD to facilitate translational studies. Alongside our cohorts of individuals with SCZ, our aim is to expand the miRNA project by integrating preclinical and clinical evidence. This integration will enable us to link specific neurophysiological phenotypes to miRNA alterations using a translational approach. The work presented here represents our initial efforts in this direction.

Finally, it has been widely hypothesized that the etiology of ASD and SCZ might be influenced by common changes occurring during brain cell development, but which then diverge biologically afterward, resulting in different clinical characteristics [21,126]. The identification of preventive strategies in preclinical approaches is particularly important in these disorders, where there is little hope for complete functional recovery once the disorder has developed. Since the set of miRNAs investigated here was assayed in the murine brain during adulthood, further research is needed to elucidate what happens in the early stages.

## 4. Materials and Methods

### 4.1. Animals and Drug Treatment: Mouse Neurodevelopmental Model for ASD and SCZ with Early NMDA Receptor Hypofunction

All experimental procedures involving animals adhered to the principles outlined in the Declaration of Helsinki and the European Communities Council Directive (86/609/EEC of 24 November 1986). These procedures were also subject to approval by the Committee of Animal Use for Research at the University Hospitals Virgen Macarena and Virgen del Rocío in Spain (project identification code: 03/09/2020/102).

The parental mouse generation consisted of four C57BL/6J male and female mice, which were obtained from Charles River Laboratories (Wilmington, MA, USA) at 8 weeks of age. Mating took place at the Ibis- Biomedicine Institute of Sevilla- CSIC facilities. Their offspring received subcutaneous injections on PNDs 7, 9, and 11, using a 25G needle, either with a vehicle—a mixture of sodium chloride (salt) and water (saline solution)—or ketamine at a dose of 30 mg/kg, as previously described by Powell et al., 2012 [127]. The ketamine used (Ketamidor 100 mg/mL, Richter Pharma, Wels, Austria) was prepared in a saline solution. This murine model of short-term NMDA receptor hypofunction was designated as ketamine-treated mice.

For all experiments, 4-month-old (±15 days) ketamine-treated mice were compared to the control group treated with the vehicle. This resulted in a total of twenty-four C57BL/6J mice, comprising both males and females, divided into two groups as follows: animals treated with the vehicle (n = 12), with 6 females and 6 males; and animals treated with ketamine (n = 12), with 6 females and 6 males. Animals whose experimental values were considered as outliers by SPSS software (version 22.0 for Windows, Chicago, IL, USA) during the statistical analysis were discarded.

### 4.2. Tissue Collection

Following anesthesia, the animals were decapitated, and PFC samples from both ketamine- and vehicle-treated mice were promptly excised, flash-frozen, and preserved at −80 °C, until utilized for miRNA profiling.

### 4.3. RNA Isolation

Briefly, total RNA was extracted from the right PFC of the ketamine and saline-treated mice using the manufacturer’s protocol (RNeasy Mini Kit, Qiagen, Hilden, Germany). The tissue was ground and homogenized using a Pellet Mixer (VWR, Radnor, PA, USA). RNA concentration was assessed in the Nanodrop ND-2000 spectrophotometer (ThermoFisher Scientific, Waltham, MA, USA) and confirmed in the Agilent 4200 TapeStation System (Agilent, Santa Clara, CA, USA), which also revealed an RNA integrity number (RIN) above 8.0 for all samples.

### 4.4. miRNA Expression Profiling

For miRNA analysis, equivalent amounts of RNA were reverse transcribed into cDNA using the miRCURY LNA RT Kit (Qiagen, Hilden, Germany). Subsequently, miRNA expression levels were determined through Real-Time Reverse Transcription-Quantitative Polymerase Chain Reaction (RT-qPCR) using Power SYBR^®^ Green PCR Master Mix (ThermoFisher Scientific, Waltham, MA, USA) in conjunction with pre-designed miRNA LNA primers obtained from Qiagen (Hilden, Germany) (please refer to Table 1 for further details).

The RT-qPCR for miRNA quantification was conducted in the QuantStudio 7 Flex RT-PCR System (Applied Biosystems, Waltham, MA, USA) with the following thermal cycling conditions: polymerase activation/denaturation and well-factor determination at 95 °C for 10 min, followed by 50 amplification cycles at 95 °C for 10 s and 60 °C for 1 min (ramp-rate 1.6 °C/s). A melt-curve analysis was carried out post-amplification to confirm the specificity of PCR products. The threshold cycle (Ct) was determined for each sample with three replicates, and the results were normalized using the housekeeping gene SNORD110. Relative expression was calculated employing the fold-change method, calculated by the 2^(−ΔΔCT)^ equation [128].

### 4.5. Statistical Analysis

For the comparison between vehicle- and ketamine-treated samples, data were analyzed using SPSS (version 22.0 for Windows, Chicago, IL, USA) and the unpaired Student’s *t*-test. Significance was established at *p* < 0.05.

The R stats package (version 4.3.1) was used to calculate the Pearson correlation coefficients. They were calculated to study the pairwise relationship between the expression levels of each of the miRNAs among experimental groups (vehicle- and ketamine-treated animals). A *p* < 0.05 was considered statistically significant. To visualize the results, the R corrplot package (version 0.92) was used to generate a Correlation Matrix (Figure 4A). Data had only one missing value, which was imputed by predictive mean matching [129] using the R mice package (version mice 3.16.0).

### 4.6. Construction of the miRNA Targeting Networks

To create the targeting network for the miRNAs, the official miRBase identifiers (IDs) for the mouse (*Mus musculus*) were interrogated using the online platform miRNet [130]. This tool is an open-source platform that comprises eleven miRNA-target prediction databases, including miRTarBase, TarBase, miRecords, SM2mir, Pharmaco-mir, mir2Disease, PhenomiR, StarBase, Epimir, miRDB, and miRanda, mainly focusing on miRNA–target interactions.

After setting both the degree and betweenness to 1.0 in the interaction table from the Network Builder menu, the target network was generated in the Network Viewer menu (Dataset S1). The analysis revealed a total of 367 nodes with a degree of at least 2 (Dataset S2). Among these, 42 nodes were common between the input 3, 4, and 5 of the miRNAs.

## 5. Conclusions

The data presented here reveal significant alterations in the PFC miRNA expression of a neurodevelopmental mouse model for ASD and SCZ induced by postnatal administration of ketamine. Specifically, we observed upregulated miR-451a and downregulated miR-137-3p. Furthermore, we identified sexual dimorphism in the expression of miR-132-3p, miR-137-3p, and miR-92a-2-5p. From a translational perspective, our preclinical results underscore the potential involvement of miR-92a-2-5p, miR-132-3p, miR-137-3p, and miR-451a in the pathophysiology of ASD and SCZ and reinforce their potential as biomarkers and therapeutic targets for these disorders. Additionally, our data emphasize the importance of considering sex as a crucial factor in researching biomarkers and developing therapeutic interventions.

## Figures and Tables

**Table 1:** List of target sequences used to determine miRNA expression in RT-qPCR. The miRNAs conserved between mice and humans are indicated.

miRNAs	Target Sequence (5′ to 3′)
hsa-miR-451a (conserved)	AAACCGUUACCAUUACUGAGUU
hsa-miR-144-3p (conserved)	UACAGUAUAGAUGAUGUACU
hsa-miR-21-5p (conserved)	UAGCUUAUCAGACUGAUGUUGA
hsa-miR-146a-5p (conserved)	UGAGAACUGAAUUCCAUGGGUU
mmu-miR-92a-2-5p (not conserved)	AGGUGGGGAUUGGUGGCAUUAC
hsa-miR-486-3p (conserved)	CGGGGCAGCUCAGUACAGGAU
hsa-miR-137-3p (conserved)	UUAUUGCUUAAGAAUACGCGUAG
hsa-miR-132-3p (conserved)	UAACAGUCUACAGCCAUGGUCG
mmu-miR-SNORD110	(Reference sequence)

hsa, Homo sapiens; miRNA(miR), microRNA; mmu, *Mus musculus*; RT-qPCR, real-time quantitative polymerase chain reaction.

## Data Availability

The datasets generated from this study are available upon request to the corresponding authors.

## Supplementary Materials

The following supporting information can be downloaded at: https://www.mdpi.com/article/10.3390/ijms25052786/s1.
