# Supplementary material for: Exploratory Analysis of MicroRNA Alterations in a Neurodevelopmental Mouse Model for Autism Spectrum Disorder and Schizophrenia
Source: Int J Mol Sci. 2024 Feb 28;25(5):2786. doi: 10.3390/ijms25052786 (PMC10932205; doi:10.3390/ijms25052786)
Supplement: Supplementary file 1 [file ijms-25-02786-s001.zip › Supplementary Table S1_final.pdf]

Supplementary Table S1. Summary of the p-values for the Pearson correlation coefficients between miRNAs.

	miR-146a-5p	miR-21-5p	miR-132-3p	miR-451a	miR-144-3p	miR-92a-2-5p	miR-486-3p	miR-137-3p
**miR-146a-5p**	**NA**	1.960	0.125	**0.016**	0.863	**0.009**	0.092	0.581
**miR-21-5p**	1.960	**NA**	0.111	**0.008**	0.878	**0.001**	**0.012**	0.604
**miR-132-3p**	0.125	0.111	**NA**	0,458	0.468	0.734	0.651	0.916
**miR-451a**	**0.016**	**0.008**	0.458	**NA**	0.857	**0.012**	0.223	0.593
**miR-144-3p**	0.863	0.878	0.468	0.857	**NA**	0.402	0.478	0.451
**miR-92a-2-5p**	**0.009**	**0.001**	0.734	**0.012**	0.402	**NA**	**0.004**	0.961
**miR-486-3p**	0.092	**0.012**	0.651	0.223	0.478	**0.004**	**NA**	0.931
**miR-137-3p**	0.581	0.604	0.916	0.593	0.451	0.961	0.931	**NA**

miRNA(miR), microRNA; NA, not aplicable.
